# Correlations of fatigue in Danish patients with rheumatoid arthritis, psoriatic arthritis and spondyloarthritis

**DOI:** 10.1371/journal.pone.0237117

**Published:** 2020-08-03

**Authors:** Bente Appel Esbensen, Sandra Elkjær Stallknecht, Maria Elmegaard Madsen, Lise Hagelund, Trine Pilgaard

**Affiliations:** 1 Copenhagen Centre for Arthritis Research, Center for Rheumatology and Spine Diseases, Centre for Head and Orthopaedics, Rigshospitalet, Glostrup, Denmark; 2 Department of Clinical Medicine, Faculty of Health and Medical Sciences, University of Copenhagen, Copenhagen, Denmark; 3 Incentive, Holte, Denmark; 4 Pfizer Denmark A/S, Ballerup, Denmark; Universita Campus Bio-Medico di Roma, ITALY

## Abstract

**Objective:**

To describe fatigue in relation to disease-specific and socioeconomic factors and to test possible correlations between fatigue and work impairment, quality of life, pain, sleep, depression, and physical functioning in people with rheumatoid arthritis (RA), psoriatic arthritis (PsA) and axial spondyloarthritis (axSpA).

**Methods:**

A questionnaire-based cross-sectional survey collecting patient characteristics such as disease characteristics, socioeconomic factors and patient-reported outcomes (PROs) from patients with RA, PsA and axSpA in Denmark. PRO scales included the FACIT-Fatigue sub-scale, Work Productivity and Activity Impairment scale (WPAI), EuroQol (EQ-5D), Medical Outcomes Study Sleep Scale (MOS), Major Depression Inventory (MDI), and Health Assessment Questionnaire (HAQ). Respondents were recruited via routine visits to the outpatient rheumatology clinic; information on diagnosis, treatment and disease activity was collected from medical journals by trained nurses.

**Results:**

487 patients participated in the study. Fatigue was more present in women, experienced patients, and patients who changed medication in the past 12 months, who were unemployed, who had less education, and who had lower household income. There was no statistically significant difference between mean fatigue in the three diagnostic groups (p = 0.08). Fatigue correlated with all included PROs (Pearson correlation coefficients, p<0.0001). Stratifying for diagnosis and adjusting for socioeconomic factors did not change the conclusion.

**Conclusion:**

In a stable, representative group of patients with RA, PsA and axSpA, we found significant correlations between fatigue and work impairment, quality of life, pain, sleep, depression and physical functioning. Fatigue cannot be perceived as a single problem, but rather as a symptom that affects broadly.

## Introduction

In healthy individuals, fatigue can occur temporarily, whereas in people with inflammatory arthritis (IA), fatigue can be persistent despite sufficient daily rest [1] and despite appropriate treatment with DMARDS (disease-modifying anti-rheumatic drugs) [2].

Fatigue is a common and a comprehensive problem for people with IA, and patients are clinically affected by their fatigue [3]. Several studies have reported prevalence of fatigue in rheumatoid arthritis (RA) ranging from 40% to 90% [3–5]. The existing literature on fatigue associated with IA is primarily based on patients with RA, and limited research has been carried out with psoriatic arthritis (PsA) and spondyloarthritis (axSpA) patients. The only study identified is a global study exploring fatigue in 30 different rheumatic diseases, which found that 41–51% of patients with RA, PsA and axSpA reported severe fatigue [6].

Fatigue is considered a multidimensional and complex phenomenon [7, 8] and is found to be associated with pain [9–12], sleep problems [13] and low physical activity [14]. Also, severe fatigue has been found to predict depression, low physical activity and quality of life, and more healthcare utilisation [15, 16]. On the other hand, depression, reduced illness beliefs, psychological distress and low self-efficacy have been found to contribute to fatigue [17, 18]. There is limited literature on the correlation between disease-specific and socioeconomic factors.

Thus, the aim of this study is first to describe fatigue in relation to disease-specific and socioeconomic factors and second to test possible correlations between fatigue and work impairment, quality of life, pain, sleep, depression and physical functioning in people with RA, PsA and axSpA.

## Materials and methods

This study was designed as a cross-sectional survey of people with RA, PsA or axSpA using validated self-reported questionnaires and medical journals. A questionnaire was developed, and data was collected using SurveyXact^®^.

Respondents who were above the age of 18 years and had a confirmed diagnosis of RA, PsA or axSpA were invited to participate in the study. We aimed for a representative sample of the IA population. Data was collected over a six-month period from January to June 2017 via rheumatology routine visits to Rigshospitalet—Glostrup and Frederiksberg. A detailed description of the methods of the study has previously been published [19].

Characteristics such as type of diagnosis, age, gender, treatment (DMARD or biologic), treatment changes and disease activity score (DAS28 for RA and PsA, BASDAI for axSpA and BASFI for PsA and axSpA) were obtained by study nurses from the respondents’ medical journals. All respondents completed a questionnaire, which included questions related to their disease history, type of household, education, employment, and household income as well as seven validated patient-reported outcomes (PROs) described in the following paragraphs.

The study comprised six standardised questionnaires covering fatigue and different outcomes related to fatigue such as work impairment, quality of life, sleep, depression and physical functioning.

*The Functional Assessment of Chronic Illness Therapy-Fatigue (FACIT-Fatigue)* was used to assess fatigue. FACIT-Fatigue is an internationally recognized disease-specific scale validated to measure fatigue in patients with a chronic illness [20]. Items in the FACIT-Fatigue scale are scored 0–4, and the total FACIT-Fatigue score range is 0–52. The higher the score, the lower the level of fatigue. *Work impairment* was measured by The Work Productivity and Activity Impairment—Specific Health Problem (WPAI-SHP) scale, a generic scale measuring work productivity and impairment in patients with specific health problems [21]. The WPAI-SHP score provides a percentage from 0% to 100% reflecting level of impairment. *Quality of Life* was measured with EQ-5D-5L, a generic instrument measuring health-related quality of life from 0 to 1, where 1 is perfect health [22]. *Sleep* was measured with The Medical Outcomes Study (MOS) sleep scale [23] on a scale of 0 to 100, where 100 indicates severe sleep problems. The Major Depression Inventory (MDI) scale was used to measure mental well-being or *depression* on a scale from 0 to 50, where <20 is normal and >29 is major depression (32). *Physical functioning* was measured by The Health Assessment Questionnaire (HAQ) [24]. HAQ assesses physical functioning as difficulty with activities of daily living over the past week, and the score ranges from 0.0 to 3.0. The HAQ-MD score used in this study is the modified DANBIO version in Danish. *Pain* was measured by a single visual analogue scale (VAS) item from the HAQ questionnaire [25]. Patients were asked “How much pain have you had in the past week?” The scale ranged from 0 to 100.

### Statistical analyses

Initially, we presented characteristics of the study population and tested variations in mean-fatigue scores between disease-specific groups and socioeconomic factors applying ANOVA. Subsequently, we assessed correlations by estimating Pearson correlation coefficients between fatigue and the six PROs: work impairment, quality of life, sleep problems, depression, physical functioning and pain. A Pearson correlation coefficient of more than 0.80 or less than -0.80 indicates a strong correlation. A coefficient of 0.40 to 0.80 or -0.40 to -0.80 indicates a moderate correlation. Finally, a coefficient below 0.40 or above -0.40 indicates a weak correlation [26]. Not all respondents answered all items, so the number of respondents varies among the different PROs.

Significant correlations were assessed in linear regressions to test the associations in more detail. Linear regression analyses were applied to estimate the association between fatigue and the six other PROs. The analyses were conducted separately. Thus, we examined the association between fatigue and the six outcomes in separate regressions. The statistical significance level was set at p<0.05.

In these analyses, we estimated the raw associations between fatigue and the PROs and conducted multiple linear regressions adjusted for the following potential confounders: type of disease (RA, PsA, axSpA), gender, age (years), years since diagnosis (0–5, 6–10, 11–15, 16–20, more than 20), treatment change in the past 12 months (0, 1, 2 or more), and current treatment (bsDMARD; csDMARD; bsDMARD and csDMARD; no current treatment). Potential confounders were selected a priori. The raw associations between fatigue and the PROs are illustrated by scatter plots with fit lines (S1 Fig). We used stepwise backward selection to assess whether the associations were best described as linear, quadratic or cubic trends. In addition, we stratified the linear regressions by type of disease to examine whether the associations between fatigue and the PROs differed according to type of disease.

In sensitivity analyses, we assessed whether confounding by socioeconomic factors could have biased our results. Thus, in addition to the remaining confounders, we included the following variables: highest obtained education, type of household (married, single, etc.), employment status (employed, unemployed, retired, etc.) and total household income.

Data management and statistical analyses were conducted using SAS^®^ statistical software version 9.4 and Microsoft Excel^®^. Analyses followed the STROBE Statement Checklist for cross-sectional studies [27].

The study was conducted under approval of the Danish Data Protection Agency (j.nr. 2012-41-1199) and with approval from the Ethics Committee of the Capital Region of Denmark (Protocol 17021125). The Danish Medicines Agency was informed about the execution of the study, as per current Danish guidelines (case no. 2017071026). All respondents gave their written informed consent before study inclusion.

## Results

The study population comprised 487 patients– 292 patients with RA (60%), 85 patients with PsA (17%) and 110 patients with axSpA (23%). The mean score of fatigue was 34.4, see Table 1. Fatigue was present in all three diagnostic groups (RA: 34.9; PsA: 31.8; axSpA: 34.8); albeit, it did not differ significantly between the disease groups (p = 0.08). The majority of the total study population was women (62%), and the mean age was 53.4. Gender, age and medical treatment were significantly correlated with fatigue with women, experienced patients and patients who changed medication in the past 12 months, with all reporting a higher mean fatigue rate. On the other hand, type of IA, age and current treatment had no impact on mean fatigue.

**Table 1:** Characteristics of the study population and mean scores of fatigue measured by FACIT-Fatigue*.

		Fatigue	
	N (%)	Mean (SD)	P-value**
All	487 (100%)	34.3 (11.1)	
**Type of disease**			0.0775
RA	292 (60%)	34.9 (11)	
PsA	85 (17%)	31.8 (10.9)	
axSpA	110 (23%)	34.8 (11.4)	
Gender			0.0079
Man	185 (38%)	36 (11)	
Woman	302 (62%)	33.3 (11.1)	
**Age**			0.3347
18–39 years	91 (19%)	34 (11.4)	
40–59 years	221 (45%)	33.7 (11.9)	
60+ years	175 (36%)	35.3 (9.9)	
**Years since diagnosis**			0.0109
0–10 years	210 (43%)	32.5 (11.9)	
11–20 years	180 (37%)	35.6 (10.6)	
More than 20 years	97 (20%)	35.8 (10)	
**Treatment change in the last 12 months**			< .0001
0	384 (79%)	35.4 (10.7)	
1	92 (19%)	30 (11.9)	
2 or more	11 (2%)	31.5 (10.6)	
**Current treatment**			0.8508
bsDMARD	191 (39%)	34 (10.9)	
csDMARD	90 (18%)	33.8 (11.6)	
bsDMARD and csDMARD	203 (42%)	34.8 (11.2)	
No current treatment	3 (1%)	37 (13.7)	

Abbreviations: bsDMARD, biological treatment; csDMARD, conventional treatment; PsA, psoriatic arthritis; RA, rheumatoid arthritis; SD, standard deviation; axSpA, spondyloarthritis.

*Higher fatigue scores indicate less fatigue (range from 0–52).

**P-value for ANOVA (95% CI)

Table 2 presents the socioeconomic characteristics of the population. We found no significant difference of fatigue according to type of household. However, education, employment and income were significantly correlated with fatigue among patients who had less education, were unemployed/retired and had lower income. Those groups showed a higher mean fatigue rate.

**Table 2:** Socioeconomic characteristics* of the study population and mean scores of fatigue measured by FACIT-Fatigue**.

		Fatigue	
	N (%)	Mean (SD)	P-value****
All	487 (100%)	34.3 (11.1)	
**Type of household**			0.0845
Married/living together	311 (64%)	35.1 (11.0)	
Living alone	162 (33%)	32.6 (11.4)	
Living at home with parents or house share with other adults	13 (3%)	34.5 (9.2)	
**Highest obtained education**			0.003
Elementary school or high school	99 (20%)	33.4 (10.3)	
Secondary or short cycle tertiary	166 (34%)	32.4 (11.6)	
Bachelor or higher	222 (46%)	36.1 (10.9)	
**Employment status**			< .0001
Employed	281 (58%)	35.7 (11.0)	
Unemployed/retired	19 (4%)	24.2 (11.9)	
Retired	123 (25%)	34.0 (9.9)	
Other***	61 (13%)	31.9 (12.2)	
**Total household income**			< .0001
Below 400.000 DKK	183 (38%)	32.5 (11.1)	
400.000 DKK or more	250 (52%)	36.5 (10.9)	
Don’t want to answer	51 (11%)	30.1 (10.2)	

*Som e socioeconomic information was not available for all respondents.

**Higher fatigue scores indicate less fatigue (range from 0–52).

***Homemaker, student or other

****P-value for ANOVA (95% CI)

**Fig 1:**
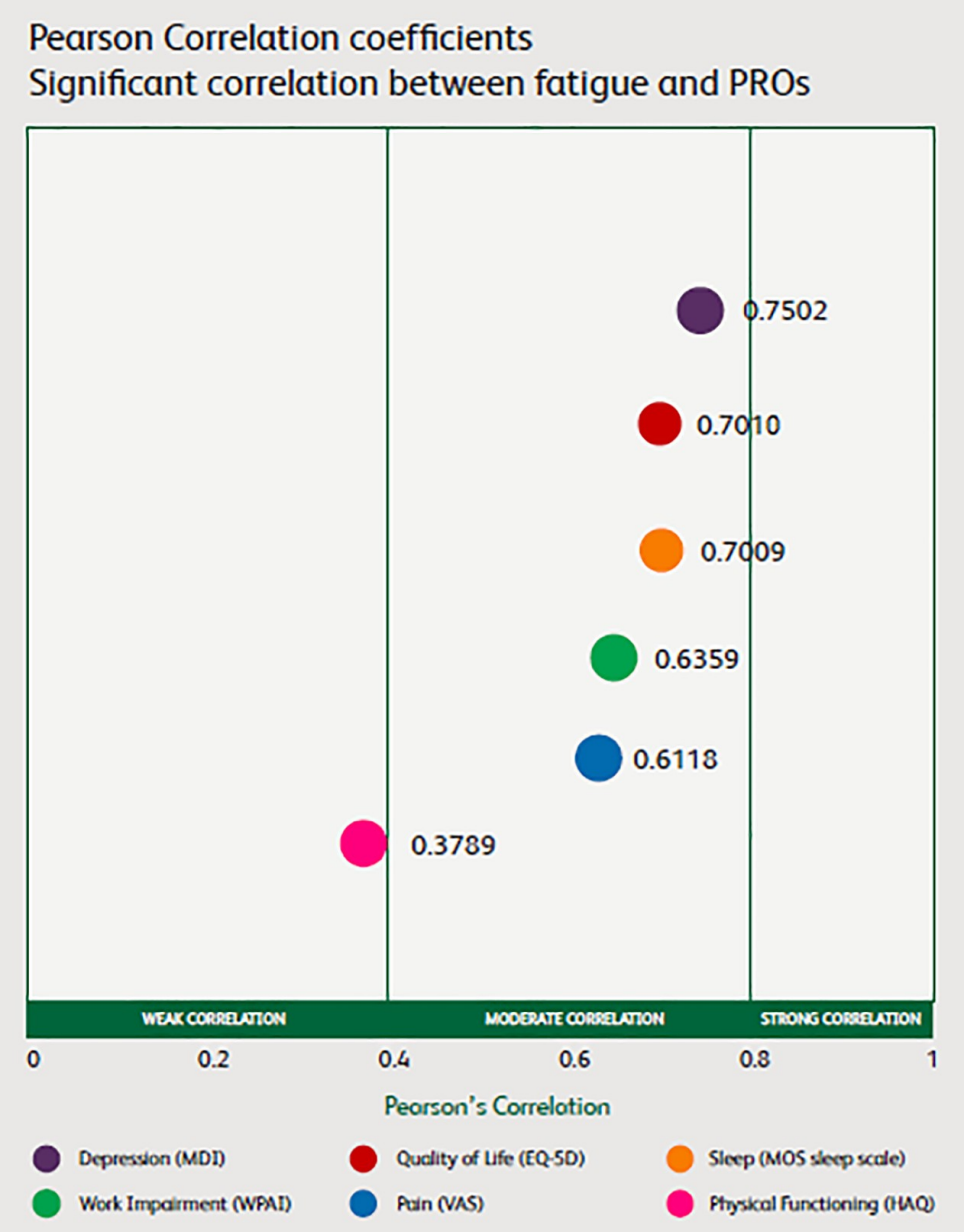
Pearson correlation coefficients between fatigue and PROs.

Fig 1 presents the Pearson correlation coefficients between fatigue and work impairment, quality of life, sleep, depression, physical functioning and pain, respectively. All correlations have a p-value of <0.0001.

Table 3 presents raw and adjusted linear regressions between fatigue and work impairment, quality of life, pain, sleep, depression and physical functioning. All six regressions show significant associations. For the adjusted association between fatigue and work impairment, the percentage of overall work impairment decreased by 1.4194% when the fatigue score increased by 1 (i.e. less fatigue). The association between fatigue and quality of life was best described as a quadratic trend. Thus, the association was strongest among patients who were suffering the most from fatigue, and the association decreased slightly when the fatigue score increased (fatigue β-value: 0.0232, fatigue^2^ β-value: -0.0002). The associations between fatigue and sleep problems and fatigue and depression were also best described as quadratic trends. The association between fatigue and sleep problems was strongest among patients who were suffering the most from fatigue, and the association increased slightly when the fatigue score increased (fatigue β-value: 0.1529, fatigue^2^ β-value: 0.0067). The association between fatigue and depression was strongest among patients who were suffering the most from fatigue, and the association decreased slightly when the fatigue score increased (fatigue β-value: -0.9560, fatigue^2^ β-value: 0.0052). The associations between fatigue and physical functioning and fatigue and pain were best described as linear trends. The risk of physical disability decreased by -0.0286 when fatigue increased by 1, and the risk of pain decreased by -1.3835 when fatigue increased by 1.

**Table 3:** Means and raw and adjusted linear regressions for the association between fatigue and work impairment, quality of life, sleep problems, depression, physical functioning and pain, respectively.

		Raw regressions			Adjusted regressions*		
Outcome (N)	Mean score (SD)	β-value	Standard error	p-value	β-value	Standard error	p-value
Work impairment (280)	23.5 (24.9)						
Fatigue		-1.4556	0.1060	< .0001	-1.4194	0.1125	< .0001
Quality of life (487)	0.79 (0.2)						
Fatigue		0.0226	0.0029	< .0001	0.0232	0.0029	< .0001
Fatigue^2^		-0.0002	0.0000	< .0001	-0.0002	0.0000	< .0001
Sleep problems (481)	44.1 (9.4)						
Fatigue		0.2002	0.1494	0.1810	0.1529	0.1483	0.3032
Fatigue^2^		0.0060	0.0023	0.0085	0.0067	0.0023	0.0033
Depression (483)	11.9 (9.3)						
Fatigue		-1.0609	0.1415	< .0001	-0.9560	0.1380	< .0001
Fatigue^2^		0.0067	0.0021	0.0020	0.0052	0.0021	0.0128
Physical functioning (483)	0.9 (0.9)						
Fatigue		-0.0302	0.0034	< .0001	-0.0286	0.0031	< .0001
Pain (482)	29.6 (25.8)						
Fatigue		-1.4127	0.0834	< .0001	-1.3835	0.0857	< .0001

*Adjusted for type of disease (RA, PsA, axSpA), gender, age (in years), years since diagnosis (0–5, 6–10, 11–15, 16–20, more than 20), treatment change in the past 12 months (0, 1, 2 or more) and current treatment (bsDMARD, csDMARD, bsDMARD and csDMARD, no current treatment).

Results from six different linear regressions between fatigue and each of the PROs. Higher scores for FACIT-Fatigue, EQ-5D and MOS sleep scale indicate better health. Lower scores for WPAI, MDI, HAQ and VAS pain from HAQ indicate better health.

The β-value indicates the slope. This means that when the fatigue score increases by 1, the outcome variable increases/decreases with the value of β.

β-values for associations between fatigue and the six other PROs did not change significantly after additional adjustment for socioeconomic factors (S1 Table). We found statistically significant correlations between fatigue and the six other PROs in both RA, PsA and axSpA patients (S2–S4 Tables). None of the stratified analyses differed markedly from the primary non-stratified analysis presented in Table 3.

## Discussion

The study shows that the mean fatigue score was considerably lower (34.4) than previously measured in the general population (44.0) [28], which underlines the severity of the problem in patients with IA. Significantly more women experienced fatigue compared with men, which is aligned with previous research that found women with RA had significantly more fatigue compared with men [29]. The reason for this is unclear, but neurophysiological differences could explain why women may experience more sustained inflammation-related fatigue compared with men [29]. Though it might not be the only explanation, a more nuanced understanding of similarities and differences in men and women is needed.

Previous research have shown that people with RA in remission continue reporting high levels of fatigue even when optimally treated with DMARDs [10, 30, 31]. In addition, a recent meta-analysis suggests that biologic treatments have only small-to-moderate effects on fatigue [32]. However, more recent studies suggest that biological treatments could have a positive impact on fatigue [33–35] and hence no consensus has been reached about the relation between fatigue and anti-inflammatory treatment.

We found a significant higher level of fatigue in the respondents who had treatment changes within the past 12 months. Changes in treatment are based on the patient’s overall condition; that is, both objective parameters and the patient’s subjective feedback. Therefore, the obvious conclusion is that patients who change treatment present with several symptoms such as pain and fatigue.

In the analysis of socioeconomic characteristics, we found a significant higher level of fatigue in respondents with less education, respondents who were unemployed/retired and respondents with a lower household income. Socioeconomic status is a well-known predictor for health outcomes, and consequently a higher mortality rate is seen in populations with low socioeconomic status [36]. Low socioeconomic status is shown to be associated with obesity and unhealthy behaviours such as smoking, heavy alcohol use and physical inactivity [37]. Patients with RA have up to a threefold risk of mortality compared with the general population [38], and a systematic review has shown that low socioeconomic status is associated with an increased risk of mortality and functional disability and risk for developing IA [39]. It is important not only for rheumatologists but for all health professionals to be aware of patients with widespread fatigue and whether patients also have low socioeconomic status, because they are expected to be more vulnerable.

The study also showed a correlation between fatigue and work impairment, quality of life, pain, sleep problems, depression and physical functioning, suggesting a close relationship between the reporting of fatigue and a number of other clinical outcomes. These findings were further supported by raw and adjusted linear regressions where we identified the association between fatigue and work impairment, quality of life, pain, sleep problems, depression and physical functioning, respectively.

Despite recent advances in understanding the role of fatigue in IA, including findings that pain, depression and physical activity may correlate with fatigue in RA [12, 14, 40], fatigue remains a challenge in rheumatology clinical practice. Limited evidence currently exists regarding the possible relationship between fatigue in different IA diseases, socioeconomic factors and other PROs, and to our knowledge this study is one of the first to access these correlations across different disease types.

Another challenge in rheumatology practice is the absence of evidence about association between fatigue and central sensitization which may have a major impact on fatigue. A review from 2007 suggests a similarity between fatigue, fibromyalgia and a central sensitization process [41]. Given an overlap between fatigue in people with inflammatory arthritis and people with fibromyalgia, the central sensitization mechanism could explain the occurrence of fatigue in some patient and the absence in other. Though, this hypothesis still needs to be investigated in future research.

People with IA report fatigue as a key issue that rheumatologists frequently overlook [10, 42], and consequently patients often believe they should accept fatigue as a part of their condition. However, based on the complexity of fatigue as we identified, rheumatologists and other health professionals instead need not only to be aware of the impact of fatigue on the individual patient’s life, but also to focus their care specifically on the relationship of fatigue with other aspects of the patient’s everyday life.

Among clinicians there is an increased recognition of the need to manage fatigue. Some evidence is available and has proven the positive effect of psychosocial interventions and physical activity on managing fatigue in people with RA [43]–e.g. mindfulness [44]; exercise [45–47]; cognitive behavioural therapy and self-management [7, 48]–and various intervention studies with a focus on reducing and/or controlling fatigue are evolving [8, 47, 49–54]. However, these studies have mainly focused on patients with RA and have not necessarily considered fatigue as a multidimensional symptom. Our results suggest this needs to be considered in future planning of interventions.

Strengths of our study include the large overall sample size, including the three diagnostic groups within IA (RA, PsA and axSpA), as well as the data source for the patient characteristics. All data was taken from the respondents’ medical journals and recorded by trained study nurses, which excludes potential recall biases seen in other surveys exclusively based on patient-reported data. The recruitment method has ensured a high response rate, and our study is considered representative of the Danish RA, PsA and axSpA populations.

Some limitations of this study should be recognized. The study cohort is slightly older and consists of more women, despite a good representation in the study cohort of both gender and age groups. The study cohort is limited to two clinics in the Capital Region of Denmark, and consequently the geographical scope is limited. Moreover, people with RA represent the majority of the study population and future research should focus on larger disease-specific populations to further investigate fatigue in people with PsA and axSpA. The nature of a cross-sectional study is that we cannot make conclusions concerning causality. It can generate only hypotheses, which can be further analysed in future studies. In this study, we focused on measuring the severity of fatigue using FACIT-Fatigue and VAS fatigue scores, which measured one aspect of fatigue (unidimensional) and not the multidimensional aspects of fatigue, which can be viewed as a limitation.

## Conclusion

Based on our study, there are number of socioeconomic factors that might be relevant to screen for when treating patients with RA, PsA and axSpA. Also, a more nuanced understanding of similarities and differences between men and women may inform future interventions for fatigue in patients with IA. To conclude, in a stable, representative group of patients with IA, we found significant correlations between fatigue and work impairment, quality of life, pain, sleep, depression and physical functioning. Fatigue cannot be seen as a single problem, but rather as a symptom that broadly affects the lives of people with IA.

## Supporting information

S1 Fig Scatterplots with fit-lines for the associations between FACIT-Fatigue and WPAI, EQ-5D, MOS sleep scale, MDI, HAQ and pain from HAQ. (TIF)

S1 Table Adjusted linear regressions for the association between fatigue and work impairment, quality of life, sleep problems, depression, physical functioning and pain, respectively, with additional adjustment for socioeconomic factor. *Adjusted for type of disease (RA, PsA, axSpA), gender, age (in years), years since diagnosis (0–5, 6–10, 11–15, 16–20, more than 20), treatment change in the past 12 months (0, 1, 2 or more) and current treatment (bsDMARD, csDMARD, bsDMARD and csDMARD, no current treatment) in addition to: highest obtained education (elementary school or high school, Secondary or short cycle tertiary, etc.), type of household (married, living alone, etc.), employment status (employed, unemployed, etc.) and total household income (below 400.000 DKK, 400.000 DKK or more, don’t want to answer). Results from six different linear regressions between fatigue and each of the PROs. Higher scores for FACIT-Fatigue, EQ-5D and MOS sleep scale indicate better health. Lower scores for WPAI, MDI, HAQ and VAS pain from HAQ indicate better health.
(DOCX)

S2 Table Raw and adjusted linear regressions for the association between fatigue and work impairment, quality of life, sleep problems, depression, physical functioning and pain, respectively, among patients with RA. *Adjusted for gender, age (in years), years since diagnosis (0–5, 6–10, 11–15, 16–20, more than 20), treatment change in the past 12 months (0, 1, 2 or more) and current treatment (bsDMARD, csDMARD, bsDMARD and csDMARD, no current treatment). Results from six different linear regressions between FACIT-Fatigue and each of the PROs. Higher scores for FACIT-Fatigue, EQ-5D and MOS sleep scale indicate better health. Lower scores for WPAI, MDI, HAQ and VAS pain from HAQ indicate better health.
(DOCX)

S3 Table Raw and adjusted linear regressions for the association between fatigue and work impairment, quality of life, sleep problems, depression, physical functioning and pain, respectively, among patients with PsA. *Adjusted for gender, age (in years), years since diagnosis (0–5, 6–10, 11–15, 16–20, more than 20), treatment change in the past 12 months (0, 1, 2 or more) and current treatment (bsDMARD, csDMARD, bsDMARD and csDMARD, no current treatment). Results from six different linear regressions between FACIT-Fatigue and each of the PROs. Higher scores for FACIT-Fatigue, EQ-5D and MOS sleep scale indicate better health. Lower scores for WPAI, MDI, HAQ and VAS pain from HAQ indicate better health.
(DOCX)

S4 Table Raw and adjusted linear regressions for the association between fatigue and work impairment, quality of life, sleep problems, depression, physical functioning and pain, respectively, among patients with axSpA. *Adjusted for gender, age (in years), years since diagnosis (0–5, 6–10, 11–15, 16–20, more than 20), treatment change in the past 12 months (0, 1, 2 or more) and current treatment (bsDMARD, csDMARD, bsDMARD and csDMARD, no current treatment). Results from six different linear regressions between FACIT-Fatigue and each of the PROs. Higher scores for FACIT-Fatigue, EQ-5D and MOS sleep scale indicate better health. Lower scores for WPAI, MDI, HAQ and VAS pain from HAQ indicate better health.
(DOCX)
