# Supplementary figures and images for: Correlations of fatigue in Danish patients with rheumatoid arthritis, psoriatic arthritis and spondyloarthritis
Source: PLoS One. 2020 Aug 3;15(8):e0237117. doi: 10.1371/journal.pone.0237117 (PMC7398515; doi:10.1371/journal.pone.0237117)

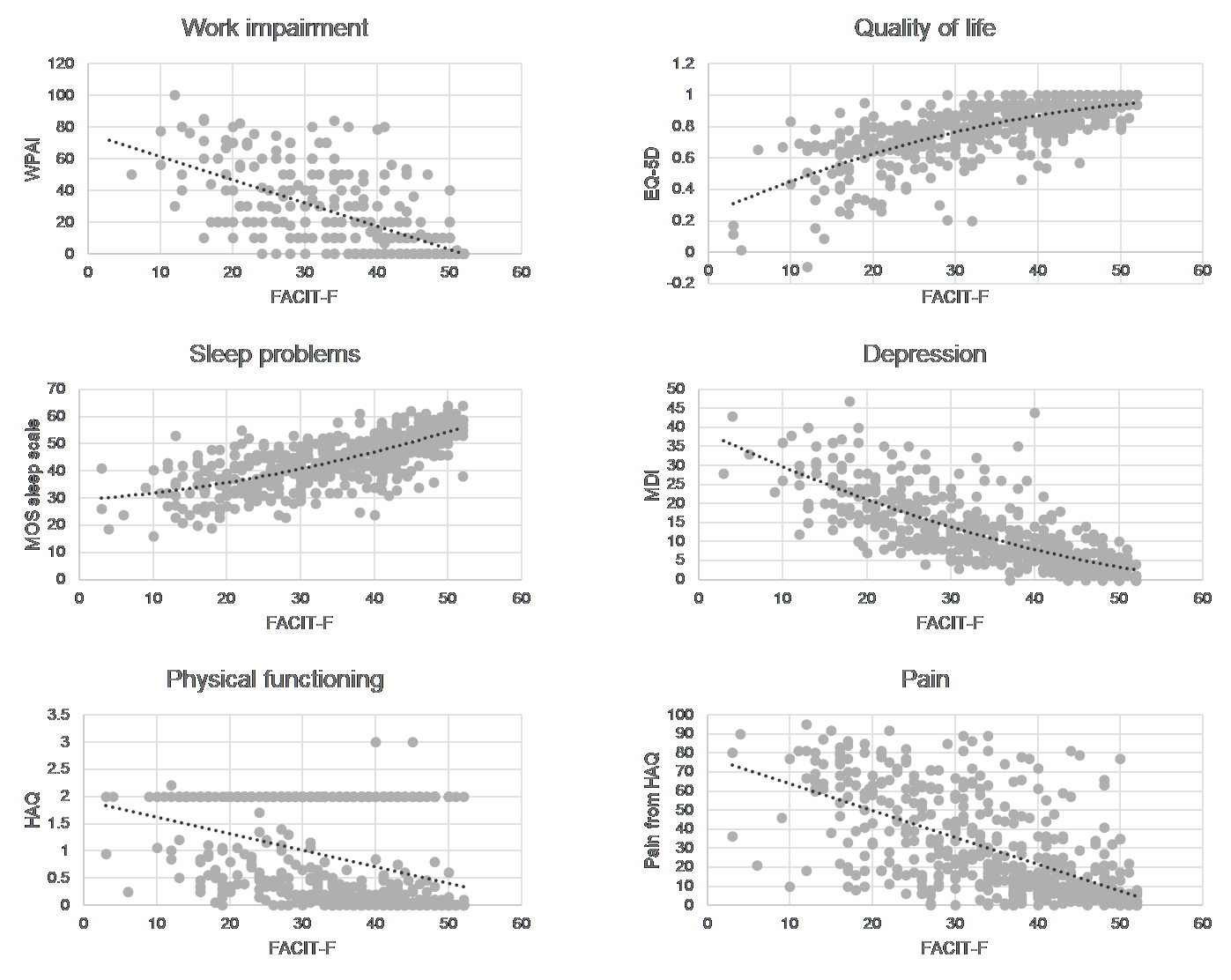

Supplement: S1 Fig — (TIF) [file pone.0237117.s001.tif]
