# Supplementary material for: Correlations of fatigue in Danish patients with rheumatoid arthritis, psoriatic arthritis and spondyloarthritis
Source: PLoS One. 2020 Aug 3;15(8):e0237117. doi: 10.1371/journal.pone.0237117 (PMC7398515; doi:10.1371/journal.pone.0237117)
Supplement: S1 Table — *Adjusted for type of disease (RA, PsA, axSpA), gender, age (in years), years since diagnosis (0–5, 6–10, 11–15, 16–20, more than 20), treatment change in the past 12 months (0, 1, 2 or more) and current treatment (bsDMARD, csDMARD, bsDMARD and csDMARD, no current treatment) in addition to: highest obtained education (elementary school or high school, Secondary or short cycle tertiary, etc.), type of household (married, living alone, etc.), employment status (employed, unemployed, etc.) and total household income (below 400.000 DKK, 400.000 DKK or more, don’t want to answer). Results from six different linear regressions between fatigue and each of the PROs. Higher scores for FACIT-Fatigue, EQ-5D and MOS sleep scale indicate better health. Lower scores for WPAI, MDI, HAQ and VAS pain from HAQ indicate better health. (DOCX) [file pone.0237117.s002.docx]

**S1 Table.** Adjusted linear regressions for the association between fatigue and work impairment, quality of life, sleep problems, depression, physical functioning and pain, respectively, with additional adjustment for socioeconomic factor.

|  |  | **Adjusted regressions*** | | |
| --- | --- | --- | --- | --- |
| Outcome (measure) | N | β-value | Standard error | p-value |
| Work impairment (WPAI) | 279 |  |  |  |
| Fatigue |  | -1.3680 | 0.1128 | <.0001 |
| Quality of life (EQ-5D) | 481 |  |  |  |
| Fatigue |  | 0.0208 | 0.0028 | <.0001 |
| Fatigue^2^ |  | -0.0002 | 0.0000 | 0.0002 |
| Sleep problems (MOS sleep scale) | 477 |  |  |  |
| Fatigue |  | 0.1558 | 0.1493 | 0.2972 |
| Fatigue^2^ |  | 0.0066 | 0.0023 | 0.0041 |
| Depression (MDI) | 479 |  |  |  |
| Fatigue |  | -0.9517 | 0.1401 | <.0001 |
| Fatigue^2^ |  | 0.0053 | 0.0021 | 0.0131 |
| Physical functioning (HAQ) | 477 |  |  |  |
| Fatigue |  | -0.0266 | 0.0031 | <.0001 |
| Pain (VAS pain from HAQ) | 479 |  |  |  |
| Fatigue |  | -1.3448 | 0.0891 | <.0001 |

*Adjusted for type of disease (RA, PsA, axSpA), gender, age (in years), years since diagnosis (0-5, 6-10, 11-15, 16-20, more than 20), treatment change in the past 12 months (0, 1, 2 or more) and current treatment (bsDMARD, csDMARD, bsDMARD and csDMARD, no current treatment) in addition to: highest obtained education (elementary school or high school, Secondary or short cycle tertiary, etc.), type of household (married, living alone, etc.), employment status (employed, unemployed, etc.) and total household income (below 400.000 DKK, 400.000 DKK or more, don’t want to answer).
Note: Results from six different linear regressions between fatigue and each of the PROs. Higher scores for FACIT-Fatigue, EQ-5D and MOS sleep scale indicate better health. Lower scores for WPAI, MDI, HAQ and VAS pain from HAQ indicate better health.
