# Supplementary material for: Correlations of fatigue in Danish patients with rheumatoid arthritis, psoriatic arthritis and spondyloarthritis
Source: PLoS One. 2020 Aug 3;15(8):e0237117. doi: 10.1371/journal.pone.0237117 (PMC7398515; doi:10.1371/journal.pone.0237117)
Supplement: S3 Table — *Adjusted for gender, age (in years), years since diagnosis (0–5, 6–10, 11–15, 16–20, more than 20), treatment change in the past 12 months (0, 1, 2 or more) and current treatment (bsDMARD, csDMARD, bsDMARD and csDMARD, no current treatment). Results from six different linear regressions between FACIT-Fatigue and each of the PROs. Higher scores for FACIT-Fatigue, EQ-5D and MOS sleep scale indicate better health. Lower scores for WPAI, MDI, HAQ and VAS pain from HAQ indicate better health. (DOCX) [file pone.0237117.s004.docx]

**S3 Table.** Raw and adjusted linear regressions for the association between fatigue and work impairment, quality of life, sleep problems, depression, physical functioning and pain, respectively, among patients with PsA.

|  |  | **Raw regressions** | | | **Adjusted regressions*** | | |
| --- | --- | --- | --- | --- | --- | --- | --- |
| Outcome (measure) | N | β-value | Standard error | p-value | β-value | Standard error | p-value |
| Work impairment (WPAI) | 48 |  |  |  |  |  |  |
| Fatigue |  | -1.5892 | 0.2282 | <.0001 | -1.2673 | 0.2460 | <.0001 |
| Quality of life (EQ-5D) | 85 |  |  |  |  |  |  |
| Fatigue |  | 0.0128 | 0.0013 | <.0001 | 0.0125 | 0.0014 | <.0001 |
| Sleep problems (MOS sleep scale) | 85 |  |  |  |  |  |  |
| Fatigue |  | 0.5408 | 0.0738 | <.0001 | 0.4927 | 0.0693 | <.0001 |
| Depression (MDI) | 85 |  |  |  |  |  |  |
| Fatigue |  | -0.6739 | 0.0653 | <.0001 | -0.6770 | 0.0671 | <.0001 |
| Physical functioning (HAQ) | 84 |  |  |  |  |  |  |
| Fatigue |  | -0.0409 | 0.0070 | <.0001 | -0.0325 | 0.0055 | <.0001 |
| Pain (VAS pain from HAQ) | 83 |  |  |  |  |  |  |
| Fatigue |  | -1.5408 | 0.2184 | <.0001 | -1.5342 | 0.2295 | <.0001 |

*Adjusted for gender, age (in years), years since diagnosis (0-5, 6-10, 11-15, 16-20, more than 20), treatment change in the past 12 months (0, 1, 2 or more) and current treatment (bsDMARD, csDMARD, bsDMARD and csDMARD, no current treatment).
Note: Results from six different linear regressions between FACIT-Fatigue and each of the PROs. Higher scores for FACIT-Fatigue, EQ-5D and MOS sleep scale indicate better health. Lower scores for WPAI, MDI, HAQ and VAS pain from HAQ indicate better health.
