# Supplementary material for: Correlations of fatigue in Danish patients with rheumatoid arthritis, psoriatic arthritis and spondyloarthritis
Source: PLoS One. 2020 Aug 3;15(8):e0237117. doi: 10.1371/journal.pone.0237117 (PMC7398515; doi:10.1371/journal.pone.0237117)
Supplement: S4 Table — *Adjusted for gender, age (in years), years since diagnosis (0–5, 6–10, 11–15, 16–20, more than 20), treatment change in the past 12 months (0, 1, 2 or more) and current treatment (bsDMARD, csDMARD, bsDMARD and csDMARD, no current treatment). Results from six different linear regressions between FACIT-Fatigue and each of the PROs. Higher scores for FACIT-Fatigue, EQ-5D and MOS sleep scale indicate better health. Lower scores for WPAI, MDI, HAQ and VAS pain from HAQ indicate better health. (DOCX) [file pone.0237117.s005.docx]

**S4 Table.** Raw and adjusted linear regressions for the association between fatigue and work impairment, quality of life, sleep problems, depression, physical functioning and pain, respectively, among patients with axSpA.

|  |  | **Raw regressions** | | | **Adjusted regressions*** | | |
| --- | --- | --- | --- | --- | --- | --- | --- |
| Outcome (measure) | N | β-value | Standard error | p-value | β-value | Standard error | p-value |
| Work impairment (WPAI) | 83 |  |  |  |  |  |  |
| Fatigue |  | -1.3836 | 0.2185 | <.0001 | -1.4216 | 0.2452 | <.0001 |
| Quality of life (EQ-5D) | 110 |  |  |  |  |  |  |
| Fatigue |  | 0.0107 | 0.0009 | <.0001 | 0.0106 | 0.0010 | <.0001 |
| Sleep problems (MOS sleep scale) | 110 |  |  |  |  |  |  |
| Fatigue |  | 2.5522 | 0.9654 | 0.0094 | 2.8588 | 0.9703 | 0.004 |
| Fatigue^2^ |  | -0.0772 | 0.0324 | 0.0189 | -0.0874 | 0.0326 | 0.0085 |
| Fatigue^3^ |  | 0.0009 | 0.0003 | 0.0096 | 0.0010 | 0.0003 | 0.0043 |
| Depression (MDI) | 109 |  |  |  |  |  |  |
| Fatigue |  | -0.7088 | 0.0520 | <.0001 | -0.6813 | 0.0559 | <.0001 |
| Physical functioning (HAQ) | 109 |  |  |  |  |  |  |
| Fatigue |  | -0.0198 | 0.0056 | 0.0006 | -0.0177 | 0.0060 | 0.0037 |
| Pain (VAS pain from HAQ) | 110 |  |  |  |  |  |  |
| Fatigue |  | -1.4332 | 0.1648 | <.0001 | -1.3472 | 0.1772 | <.0001 |

*Adjusted for gender, age (in years), years since diagnosis (0-5, 6-10, 11-15, 16-20, more than 20), treatment change in the past 12 months (0, 1, 2 or more) and current treatment (bsDMARD, csDMARD, bsDMARD and csDMARD, no current treatment).
Note: Results from six different linear regressions between FACIT-Fatigue and each of the PROs. Higher scores for FACIT-Fatigue, EQ-5D and MOS sleep scale indicate better health. Lower scores for WPAI, MDI, HAQ and VAS pain from HAQ indicate better health.
